# Persistent intra-abdominal abscess with intestinal obstruction following seven failed drainage procedures over 3.5 years: a case report

**DOI:** 10.1186/s12893-025-03314-9

**Published:** 2025-12-05

**Authors:** Ayman Shemes, Salma Samra, Ahmed Mohamed, Amr A. Elgharib

**Affiliations:** 1 Department of General Surgery, Mansoura University-Faculty of Medicine-Egypt, Mansoura, Egypt; 2 Intern Doctor, Mansoura University-Faculty of Medicine-Egypt, Mansoura, Egypt

**Keywords:** Intra-abdominal abscess, Post-operative infections, Intestinal obstruction, Case report

## Abstract

**Background:**

Intra-abdominal abscesses are common post-operative complications, typically resolving with percutaneous drainage and antibiotics. Chronic, refractory abscesses persisting for years and progressing to mechanical bowel obstruction are exceedingly rare.

**Case Presentation:**

We report a 36-year-old female who developed a persistent intra-abdominal abscess following an ovarian cystectomy, with symptoms persisting for 3½ years despite seven drainage procedures and two exploratory laparotomies. She presented with progressive abdominal distension, vomiting, and signs of bowel obstruction. Imaging confirmed a chronic organized abscess abutting bowel loops. Definitive management included bowel and omental resection with restoration of bowel continuity. Histopathology revealed chronic suppurative inflammation without granulomas or malignancy.

**Conclusion:**

This case highlights the need to consider chronic intra-abdominal abscess in the differential diagnosis of persistent post-operative symptoms and underscores the importance of timely surgical intervention in refractory cases to prevent progression to rare complications such as mechanical bowel obstruction.

## Introduction

 Post-operative intra-abdominal abscesses are well-recognized complications of abdominal surgery, often managed successfully with image-guided percutaneous drainage and antimicrobial therapy. According to the World Society of Emergency Surgery (WSES) guidelines, early recognition, effective source control, and appropriate antibiotic therapy are critical to reducing morbidity and mortality associated with intra-abdominal infections. In most cases, abscesses resolve with one or two drainage procedures, minimizing the need for extensive surgical intervention [1].

However, cases of chronic, refractory post-operative intra-abdominal abscesses persisting for more than two years are exceptionally rare [2]. Such abscesses may create a hostile intra-abdominal environment characterized by dense adhesions and chronic inflammation, occasionally progressing to mechanical bowel obstruction, an uncommon but serious complication. We present a rare case of a post-operative intra-abdominal abscess persisting for 3½ years despite multiple drainage attempts and requiring bowel resection and anastomosis, highlighting the challenges and considerations in managing this uncommon presentation.

### Patient information

A 36-year-old married female presented to the General Surgery Outpatient Clinic at Mansoura University Hospitals with persistent vomiting and diffuse abdominal pain. She had no significant past medical, familial, or psychosocial history.

Approximately three and a half years prior to presentation, the patient had undergone a left ovarian cystectomy at another institution due to a midline abdominal swelling accompanied by lower abdominal pain. The procedure was uneventful intraoperatively. However, within a few days postoperatively, she began to experience worsening abdominal pain and clinical signs suggestive of a surgical site infection. Imaging at the time revealed a subcutaneous abscess, which was managed by percutaneous drainage.

The patient’s symptoms persisted for two months despite the initial intervention. A second drainage procedure was performed without significant symptom relief. A contrast abdominal computed tomography (CT) scan subsequently revealed superficial inflammatory collections along the surgical wound. A third drainage procedure was undertaken.

Following this, the patient continued to experience abdominal pain, increasing abdominal distension, and persistent swelling over the surgical site. A barium meal study was performed and reported as normal. Due to ongoing symptoms, another contrast abdominal computed tomography (CT) scan was done revealing non-specific superficial inflammatory collection along the lower abdominal wall. A lower-midline exploratory laparotomy was performed, revealing an abscess that was drained, and an omentectomy was completed.

Over the subsequent two years, her symptoms persisted and worsened, with progressive abdominal distension, frequent vomiting, and significant unintentional weight loss. During this period, she underwent three additional drainage procedures, all of which failed to achieve lasting symptom control.

Eight months prior to her current presentation, the patient underwent a second lower-midline exploratory laparotomy. Intraoperative findings led to an appendectomy, left oophorectomy, and bilateral salpingectomy. However, her symptoms remained unresolved, and she continued to suffer from chronic abdominal pain, intermittent vomiting, and marked abdominal distension. She was then referred to Mansoura University Hospitals for further evaluation and management (Table 1).

**Table 1:** Timeline

Timeframe	Event
0 months (July, 2021)	Left ovarian cystectomy for midline swelling
Days post-op	Developed worsening pain; subcutaneous abscess drained
+ 2 months (Oct, 2021)	Persistent symptoms; second drainage performed
+ 3 months (Nov, 2021)	Third drainage performed after CT showing superficial collection
+ 4 months (Dec, 2021)	Barium meal normal; exploratory laparotomy with fourth drainage and omentectomy
+ 4–28 months (Dec 2021- Oct 2022)	Persistent and worsening symptoms, vomiting, weight loss
+ 28 months (Oct, 2022)	Fifth, sixth, and seventh drainage procedures performed without relief
+ 40 months (May, 2024)	Second exploratory laparotomy with appendectomy, left oophorectomy, bilateral salpingectomy
+ 42 months	Persistent symptoms; referred for further evaluation
Current (Jan, 2025)	Presented with bowel obstruction and infected wound; definitive management with bowel and omental resection, abscess cavity clearance, and anastomosis

### Clinical findings

The patient presented with a clinical picture of intestinal obstruction, characterized by persistent vomiting, progressive abdominal distension, intermittent lower abdominal pain, and constipation. On examination, there was diffuse abdominal distension with localized tenderness over the lower abdomen. Bowel sounds were absent. Inspection of the abdomen revealed an infected midline surgical incision that appeared erythematous, indurated, swollen, and tender. The patient was hemodynamically unstable, with hypotension (blood pressure: 88/57 mmHg) and tachycardia (heart rate: 103 beats per minute), feverish (Tempeature: 38 celsius) consistent with systemic inflammatory response and early shock. These findings, in conjunction with her clinical history, raised suspicion of an intra-abdominal infectious process complicated by bowel obstruction.

### Diagnostic assessment

Pre-operative investigations included a barium meal study, which revealed smooth contrast flow through the lower esophagus and stomach, with no ulceration, filling defects, outpouchings, hiatus hernia, or evidence of gastroesophageal reflux disease (GERD). Abdominal ultrasound showed a small, well-defined echogenic collection measuring approximately 25 × 14 mm, located just supra-umbilically and at a depth of about 20 mm from the skin surface, suggestive of a localized inflammatory or abscess collection. Additionally, small echogenic gallbladder stones (approximately 5 mm) with mild wall thickening and minimal biliary sludge were observed, while the liver, pancreas, spleen, kidneys, uterus, and adnexae were unremarkable. A contrast abdominal C.T scan revealed Lower abdominal marginally enhancing intra-peritoneal collection with extension to anterior abdominal wall with bulk of collection measuring 5.9 × 4.2 × 6.0 cm with air densities inside and surrounding fat stranding, the collection is seen abutting the bowel loops with no definite infiltration.

### Therapeutic intervention

Upon presentation, the patient was shocked as result of the persistent vomiting and reduced oral intake. She received 2500 ml of crystalloids, one unit of plasma and one unit of packed RBCs. A urinary catheter was placed for monitoring UOP. An appropriate fluid response was achieved and the vital signs normalized within the next 24 hours.

Under general anesthesia and in the supine position, a midline exploratory laparotomy was performed. The procedure began with the excision of a 2 cm skin sinus tract. On exploration, an intra-abdominal abscess cavity containing pus was identified. Culture samples were obtained.

The abscess cavity was found to involve the omentum, a portion of the transverse colon, and a segment of the small intestine approximately 2 m distal to the duodenojejunal (DJ) junction. Resection of the affected omental tissue and involved small bowel segment was performed. Bowel continuity was restored with a two-layer, hand-sewn end-to-end anastomosis.

A pelvic drain was placed, and the abdominal wall was closed in layers, including fascial and subcutaneous closure, followed by skin closure.

Postoperative instructions included oral intake after 3 days, use of an abdominal binder for 6 weeks, and daily wound care. The patient was discharged on the sixth postoperative day in stable condition.

### Histopathological findings report

**Fig. 1:**
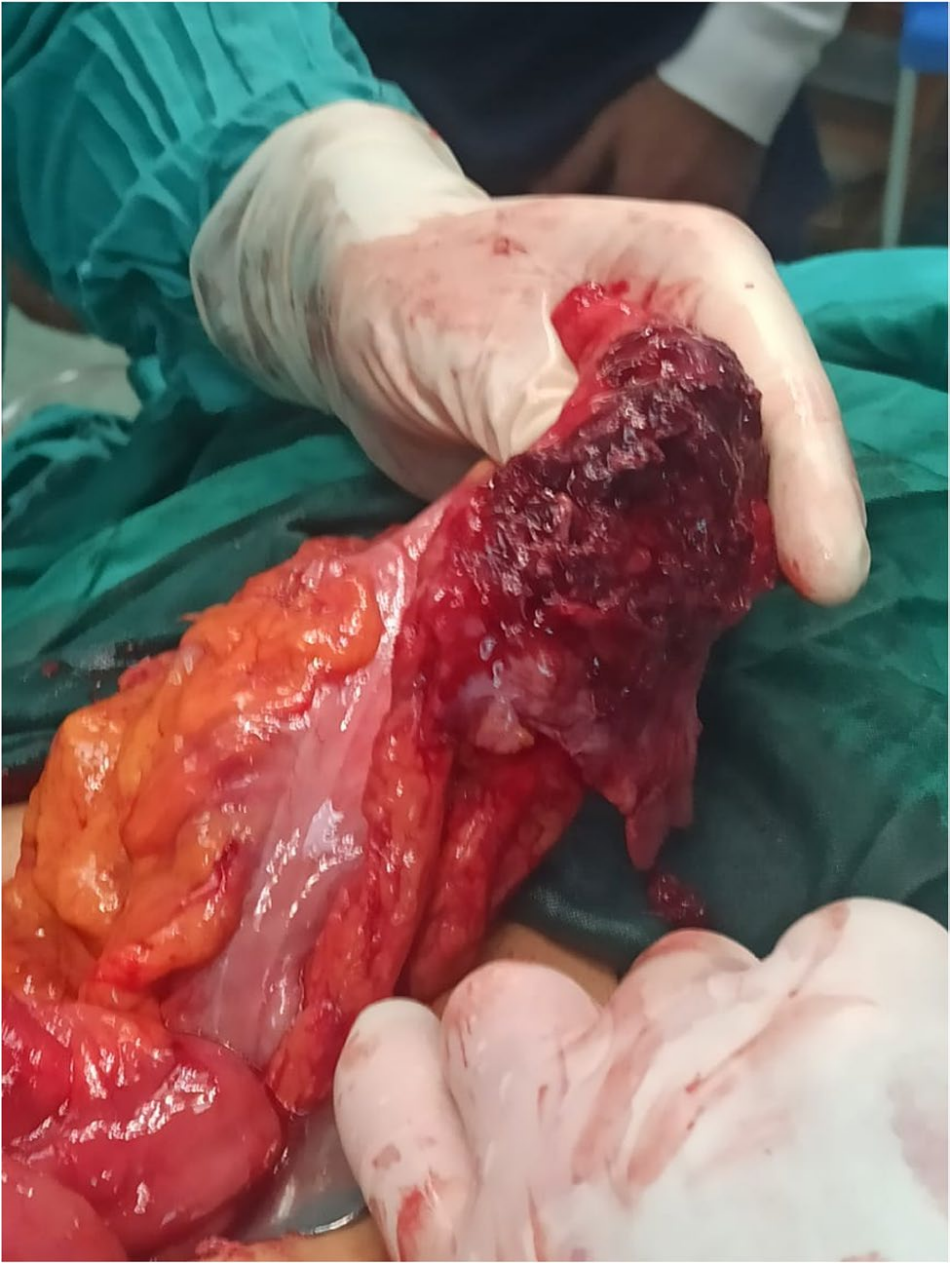
Demonstrate the involved parts of the small intestine and the omentum

**Fig. 2:**
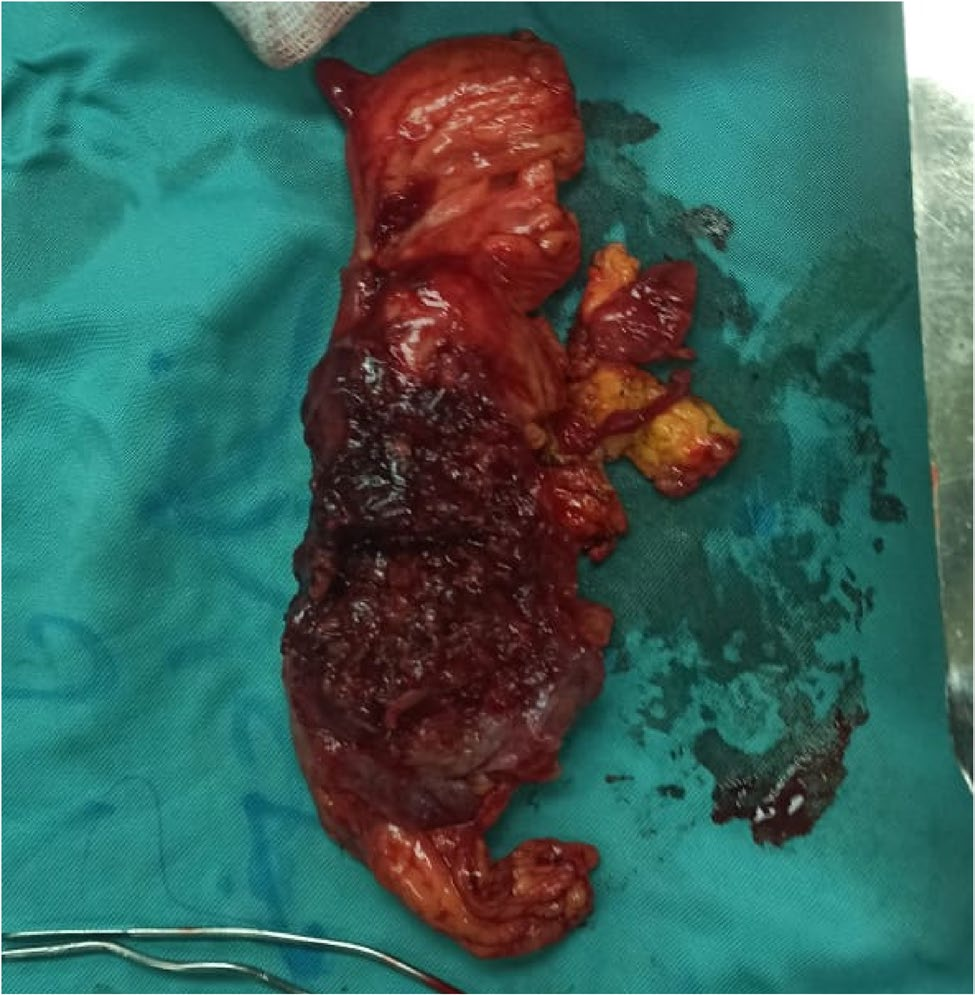
Demonstrate the involved parts of the small intestine and the omentum

The patient underwent surgical exploration for a chronic intra-abdominal collection involving the omentum of the transverse colon and a segment of small intestine approximately 2 cm distal to the duodenojejunal (DJ) junction, forming an organized abscess cavity. Two specimens were submitted for pathological examination (Figs. 1 and 2).

- Gross examination:
  Specimen (1), consisting of omental tissue, measured 12 × 3 cm and appeared grossly unremarkable; two representative tissue blocks were taken.
  Specimen (2), an unlabeled segment of small intestine measuring 10 × 3 cm, showed grossly normal mucosa on cut section, except for a friable, blackish area suggestive of an abscess. Two safety margins were identified and sampled, with representative blocks taken from the mucosa and bowel wall for histological analysis.
- Microscopic examination:
  Histology of the small intestinal specimen revealed preserved mucosal architecture. The underlying wall demonstrated focal areas of coagulative necrosis and chronic suppurative inflammation, marked by a dense mixed-cell infiltrate including neutrophils, pus cells, lymphocytes, plasma cells, and eosinophils. Vascular congestion and fibrosis were noted. One reactive lymph node was also identified. Both proximal and distal surgical margins were viable and free of pathological involvement. No granulomas, cellular atypia, or malignant features were observed.
  The omental tissue showed areas of fat necrosis accompanied by a mixed inflammatory infiltrate composed primarily of lymphocytes, neutrophils, and pus cells, along with focal congestion. No atypia or malignancy was detected.

## Discussion

This case illustrates the limitations of the stepwise approach- repeated percutaneous drainage combined with antibiotics- for managing intra‑abdominal abscesses, particularly when abscesses are organized, multiloculated, or associated with dense adhesions. Although percutaneous catheter drainage (PCD) is successful in about 80–90% of postoperative abscesses, failure is significantly more likely when abscess cavities are complex or associated with necrosis or enteric communication. The WSES guidelines similarly emphasize early, effective source control- percutaneous drainage first, but advocating prompt surgical intervention when drainage fails. In most uncomplicated postoperative abscesses, resolution typically occurs within weeks, not years, following gynecologic surgery. In contrast, this patient’s abscess persisted for over 3 years, despite seven drainage attempts and two laparotomies, representing an exceptionally protracted and rare clinical course. Chronic postoperative intra‑abdominal abscess after procedures such as ovarian cystectomy are uncommon and rarely extend over such a duration. This delay likely contributed to progression into bowel involvement and eventual mechanical obstruction. Histopathological findings demonstrated involvement of multiple structures- omentum, transverse colon, and small bowel- with coagulative necrosis and chronic suppurative inflammation, consistent with an organized, chronic abscess cavity rather than sterile fluid collection or inflammatory adhesions.

The patient’s resistance to standard treatment and failure of multiple drainage procedures, despite PCD success rates of up to ~ 90%, underscores the necessity of earlier consideration of surgical resection in refractory cases. This case also demonstrates rare complications, including intestinal obstruction, systemic inflammatory response, and sinus tract formation with abdominal wall involvement, which are unusual sequelae of gynecologic surgeries. This supports the importance of a multidisciplinary approach, including surgical, radiologic, and histopathological evaluation, and highlights the role of comprehensive histopathological assessment to exclude alternative etiologies such as malignancy or granulomatous conditions [1, 3–8].

### Pathophysiology of chronicity and multiplied failed drainages

Several interrelated factors likely contributed to the extraordinary persistence of the abscess across seven unsuccessful drainage interventions over 3.5 years:

1. Loculated abscess cavities
  Dense fibrous walls and multiple loculations may inhibit the complete evacuation of pus, rendering repeated drainage procedures ineffective. An inflammatory barrier often forms around intra-abdominal collections, confounding standard treatment approaches [9].
2. Biofilm or Organized Inflammation
  Chronic suppurative inflammation can lead to biofilm formation or organized fibrotic tissue, impeding antibiotic penetration and immune clearance—even when drainage appears successful.
3. Suboptimal source control
  Guidelines emphasize combined antibiotic therapy and adequate drainage as essential for intra-abdominal abscess resolution [10]. In this case, the persistence despite repeated attempts suggests that full source control was not achieved until definitive surgery with bowel and omental resection.
4. Hostile abdominal environment
  Repeated interventions likely created dense adhesions and a hostile abdominal milieu, making both drainage and surgical resolution more complex. Such environments are recognized risk factors for persistent intra-abdominal infections [10].

### Differential diagnosis

Persistent intra-abdominal abscesses pose a complex diagnostic challenge. Beyond malignancy and granulomatous diseases, several other etiologies merit consideration [11–13]:

- *Actinomycosis* is a rare, chronic bacterial infection that frequently mimics neoplasms or inflammatory conditions. In the abdominopelvic region, it may manifest with sinus tracts or slow-growing masses and requires histopathology or culture for confirmation.
- *Atypical mycobacterial infections* and *tuberculosis* can cause persistent abscesses with granulomatous features, although these were excluded in our patient by histology (lack of granulomas) and negative microbiology.
- *Foreign body-associated infections*, such as from retained surgical materials, may contribute to chronicity; these trigger persistent inflammation but were not evident here.

In our case, histopathology revealed suppurative inflammation without granulomas, atypia, or sulfur granules; microbiology failed to isolate organisms like *Actinomyces* or mycobacteria. Thus, alternative diagnoses were systematically ruled out.

While chronic postoperative abscesses are rare, a few reports provide useful reference points (Table 2).

**Table 2:** Reported cases in the literature with similar presentations

Reported Case	Time from Surgery	Etiology	Interventions	Outcome
Abdominal actinomycosis mimicking malignancy [12]	Variable (indolent onset)	Actinomyces	Surgical resection	Infection resolved
Appendiceal actinomycosis with inflammatory mass [14]	Chronic presentation	Actinomyces	Antibiotics/surgery	Diagnosis confirmed
General intra-abdominal abscess cohorts [10]	Postoperative	Mixed	Drainage (+ antibiotics)	Most resolved; some required surgery

#### Key differences in our case

- Exceptionally long duration (3.5 years) with ongoing symptoms.
- Seven failed drainage attempts before definitive surgery.
- Development of mechanical intestinal obstruction.
- Pathology revealed only chronic suppurative inflammation—no actinomycotic or granulomatous features.

This structured comparison highlights the unprecedented chronicity, intervention resistance, and mechanical complication of our case, underlining its novelty and clinical significance.

## Conclusion

Persistent intra-abdominal abscesses can progress to chronicity and mechanical bowel obstruction despite repeated drainage attempts, necessitating definitive surgical management. Early recognition and individualized management are critical in refractory intra-abdominal abscess cases, consistent with WSES principles emphasizing early effective source control. Histopathological examination is essential to exclude other causes such as Crohn’s disease and malignancy in chronic intra-abdominal abscesses. Chronic post-operative abscesses should be considered in the differential diagnosis of persistent abdominal symptoms, even years after surgery.

Written informed consent was obtained from the patient for publication of this case report and accompanying images. A copy of the written consent is available for review by the Editor-in-Chief of this journal upon request.

## Data Availability

Not applicable.
